# Precision Attachment in the Rehabilitation of Distal Extension Using the Functionally Generated Path (FGP) Technique: A Clinical Case Report

**DOI:** 10.7759/cureus.110868

**Published:** 2026-06-15

**Authors:** Apoorva K, Maneesha P K, PunyaKrishnan B, Mohammed Safwaan Sheikh, Pallawi Minhas

**Affiliations:** 1 Prosthodontics, Oxford Dental College and Hospital, Bangalore, IND; 2 Prosthodontics, Educare Institute of Dental Sciences, Malappuram, IND

**Keywords:** cpd - kennedy class i mod 1, distal extension, dynamic occlusion, extra coronal attachment, fgp, functional efficiency, functional occlusion, precision attachments, removable partial denture, semi-adjustable articulator

## Abstract

Removable partial dentures retained by precision attachments are a valuable alternative to conventional removable partial dentures. Ensuring the long-term success depends on adhering to the principle of widespread stress distribution and incorporating a substantial safety margin by splinting multiple teeth. Technical advancements in partial denture construction are continually being introduced, and one such advancement is precision attachment. This case report showcases the method of fabrication of mandibular partial denture using ball attachment.

## Introduction

Rehabilitation of mandibular distal extension cases, particularly Kennedy Class I situations, presents a significant challenge in prosthodontics due to the lack of posterior abutments and the resulting variation between tooth- and tissue-borne structures. Conventional clasp-retained removable partial dentures (RPD) in such cases may compromise esthetics and transmit unfavorable stresses to the abutment teeth.

Precision attachments offer a viable alternative by improving retention, stability, and patient acceptance while providing better distribution of occlusal forces. The precision attachment, a resilient extracoronal attachment, is particularly beneficial in distal extension cases as it allows controlled movement of the prosthesis, thereby reducing torque on abutment teeth and enhancing comfort [1].

To further optimize occlusal harmony, the Functionally Generated Path (FGP) technique can be employed. This technique records the patient’s functional mandibular movements, enabling the fabrication of restorations that are in precise harmony with dynamic occlusion, thus minimizing interferences and improving masticatory efficiency.

This case report describes the prosthetic rehabilitation of a mandibular Kennedy Class I mod 1 partially edentulous arch in a patient using a precision attachment in combination with the FGP technique. The approach emphasizes the integration of biomechanical principles and functional occlusion to achieve a stable, esthetic, and long-lasting prosthetic outcome [2].

## Case presentation

**Figure 1:**
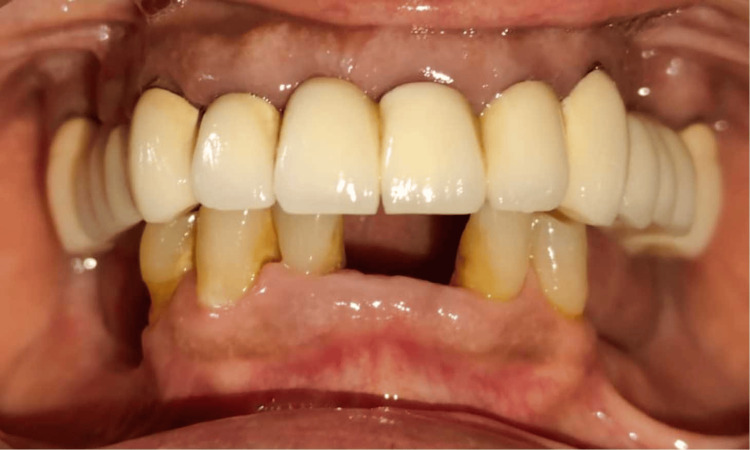
Kennedy Class I mod 1 edentulous mandibular arch and fixed partial denture on maxillary arch

A 68-year-old male patient reported to our clinic with inability to chew, seeking replacement of his missing lower teeth. Patient had a fixed partial denture in his maxillary arch (Figure 1). Clinically missing teeth are 31,34,35,36,37,41,45,46 and 47. In this case, the mandibular arches exhibited Kennedy Class I mod 1 classification (Figure 1).

He was planned for fixed partial denture for 33,32,31,41,42,43 and 44, along with precision attachment for 34,35,36,37,45,46 and 47.

**Figure 2:**
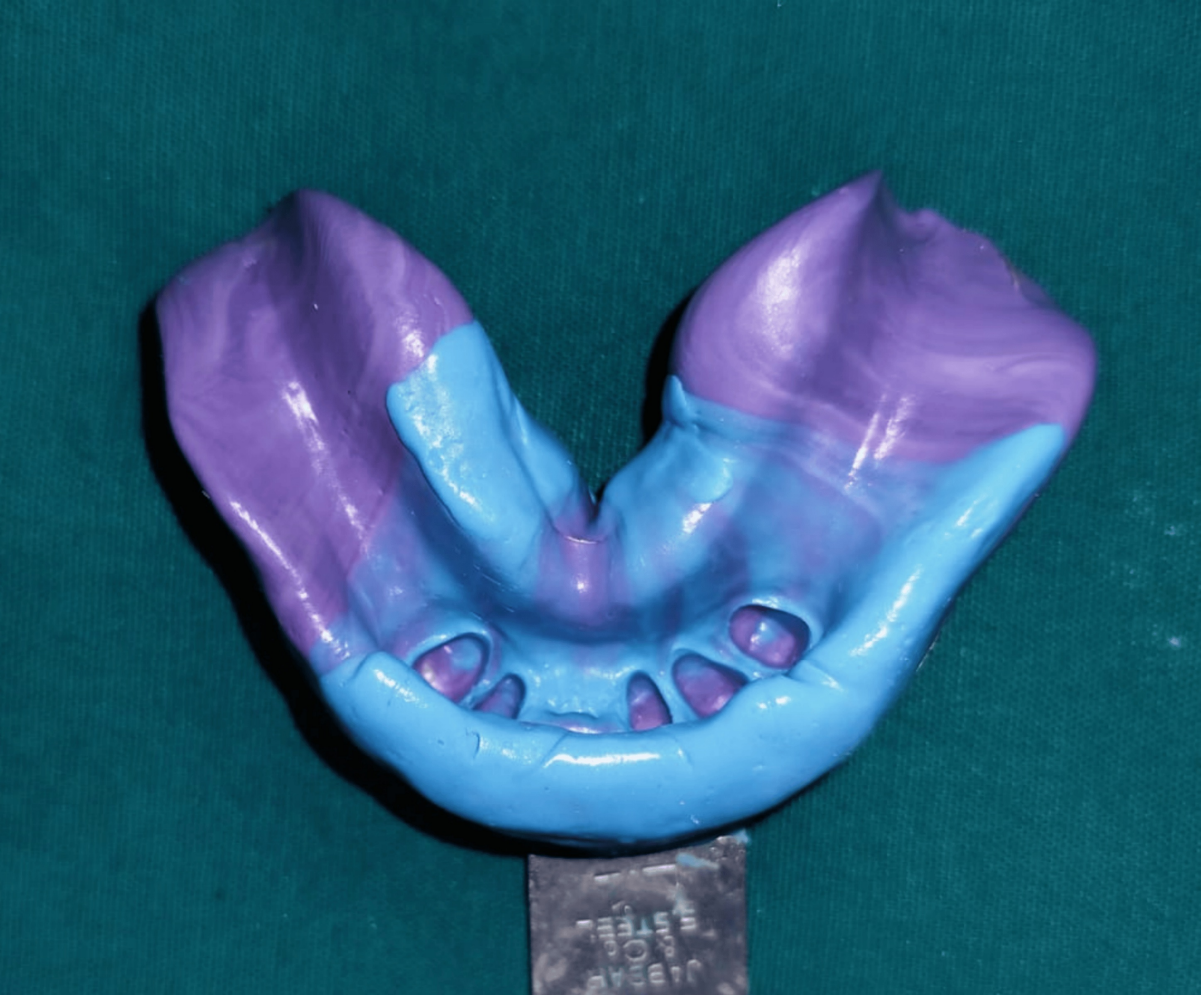
Mandibular arch impression for fixed partial denture

**Figure 3:**
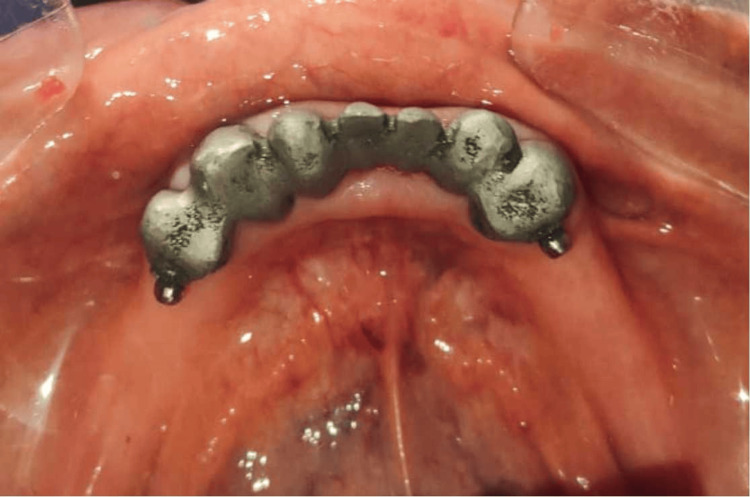
Metal trial with ball attachment

Tooth preparation was done for 33,32,42,43 and 44, followed by an impression for fixed partial denture (Figure 2). Metal trial was done with ball attachment (Figure 3), followed by cast partial denture (CPD) framework trial and bite registration. Casts were then mounted on a semi-adjustable articulator with the established vertical dimension and the RPD was designed. The FGP technique was used to arrange posterior teeth as it utilizes the patient's masticatory system to develop occlusion as it is simple, accurate and reliable.

Anterior Bisque trial and posterior RPD trial were done simultaneously and occlusion was checked, followed by selective grinding. An extra coronal ball semi-precision attachment to support posterior teeth was used. All the anterior teeth were restored with a fixed porcelain-fused-metal bridge. 

**Figure 4:**
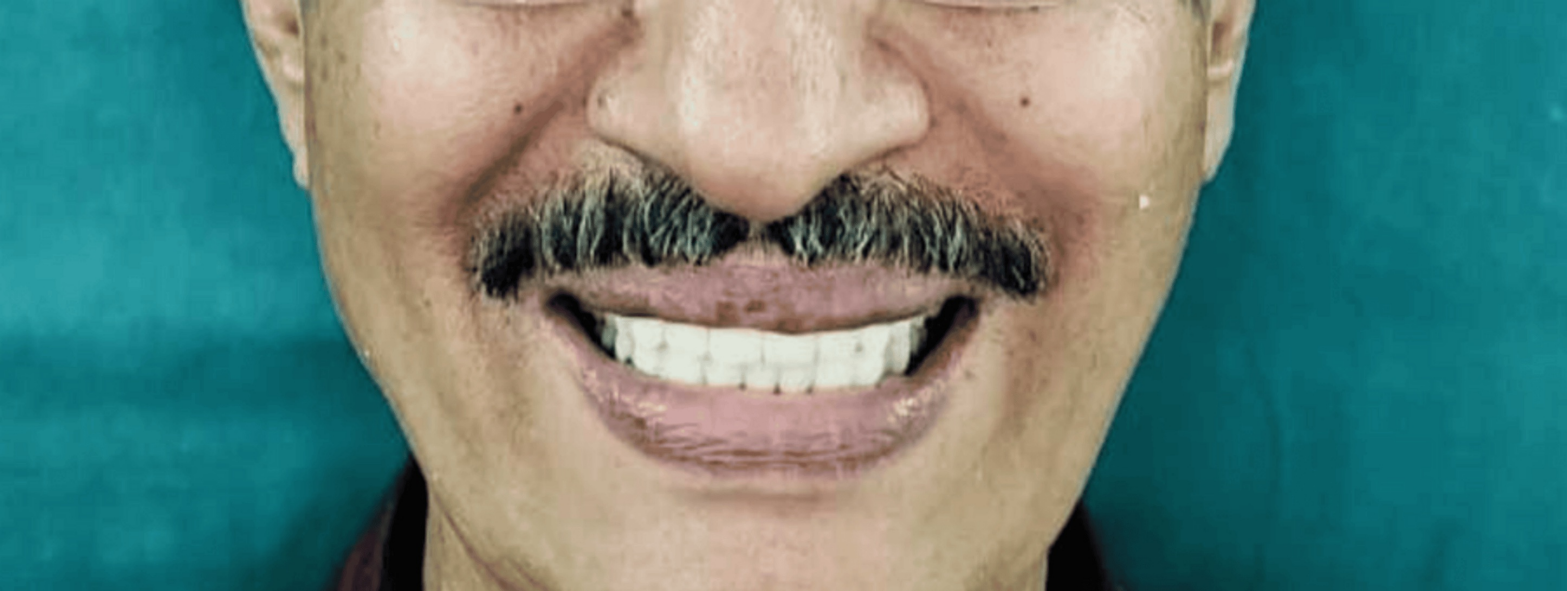
Final insertion

The final fit of CPD was evaluated and verified intraorally. Esthetics and function were improved (Figure 4).

## Discussion

Management of distal extension cases remains a prosthodontic challenge due to the absence of posterior abutments and the inherent difference in support between teeth and edentulous ridges. This disparity often results in rotational movements of the prosthesis, leading to increased stress on abutment teeth and compromised stability when conventional clasp-retained RPDs are used.

In the present case, the use of precision attachment in the mandibular arch provided a significant advantage over conventional retainers. Resilient extra coronal attachment permits limited vertical and rotational movement of the distal extension base, thereby acting as a stress breaker. This helps in distributing occlusal forces more favorably, between the abutment teeth and the residual ridge, reducing torque and preserving periodontal health. Studies have shown that the survival rates are 83.35% in five years, 67.35% in 15 years, and 50% in 20 years [3]. Additionally, the elimination of visible clasps improved the esthetic outcome and patient acceptance [4].

Another critical aspect of this case was the incorporation of the FGP technique. Accurate occlusal harmony is essential in distal extension prostheses to prevent instability and tissue irritation. The FGP technique records functional mandibular movements directly, allowing the occlusal morphology to be shaped according to the patient’s neuromuscular patterns. This reduces occlusal interferences, enhances masticatory efficiency, and contributes to the longevity of the prosthesis. 

The combined use of precision attachments and functional occlusal recording technique reflects a shift toward more biologically compatible and patient-centered prosthodontic care. However, such approach requires meticulous case selection, precise clinical execution, and adequate patient compliance. Factors such as sufficient crown height, periodontal health of abutments, and patient dexterity must be carefully evaluated before opting for precision attachments [5].

Despite the advantages, limitations such as increased cost, technique sensitivity, and the need for periodic maintenance should be considered. Long-term success also depends on regular follow-up and patient education regarding hygiene and prosthesis care.

Overall, the integration of the lower precision attachment with the FGP technique in this mandibular distal extension case demonstrated improved retention, stability, esthetics, and functional efficiency, making it a valuable treatment option in selected cases.

## Conclusions

The integration of precision attachment in mandibular arch and the FGP technique offers an effective, high-precision solution for mandibular Kennedy Class I mod 1 edentulous arch, balancing biomechanical stress-breaking with dynamic occlusion. This approach improves masticatory efficiency and aesthetics over traditional clasp-retained dentures, serving as a superior, though demanding, alternative for long-term prosthetic success.
